# Deformable lung models for anatomical lung resections: The introduction of simulated reality for imaging guidance

**DOI:** 10.1016/j.xjtc.2025.10.022

**Published:** 2025-11-10

**Authors:** Quinten J. Mank, Tjerko Kieft, Sabrina Siregar, Alexander P.W.M. Maat, Jolanda Kluin, Amir H. Sadeghi

**Affiliations:** a Department of Cardiothoracic Surgery, Thoraxcenter, Erasmus MC, University Medical Center Rotterdam, Rotterdam, The Netherlands; b Surgical Reality, Nieuw-Vennep, The Netherlands; c Heart and Lung Division, Department of Cardiothoracic Surgery, University Medical Center Utrecht, Utrecht, The Netherlands

**Keywords:** lung segmentectomy, lobectomy, deformable models, artificial intelligence

## Abstract

**Objective:**

This study introduces PulmoSimulatedReality (Pulmo-SR), a novel technique combining artificial intelligence, finite element method, 3-dimensional (3D) visualization, and 4-dimensional (4D) interaction for preoperative imaging and intraoperative surgical guidance in pulmonary resections, such as lobectomy and segmentectomy. The clinical applicability of this 3D modeling approach is evaluated through a preliminary validation protocol.

**Methods:**

A deep learning algorithm was employed to generate 3D segmentations of patient anatomy. 3D models were created for 30 patients undergoing pulmonary resection, and 4D models were developed using the Pulmo-SR platform, incorporating finite element methods for dynamic deformation. Clinical validation was conducted by assessing accuracy, precision, and sensitivity using retrospective intraoperative video recordings alongside dynamic 4D models. Latency and 3D model reconstruction time were also measured.

**Results:**

Validation of 30 cases yielded high average scores for accuracy, precision, and sensitivity, respectively: artery (0.987 ± 0.047, 0.993 ± 0.037, and 0.994 ± 0.031), vein (0.976 ± 0.099, 0.976 ± 0.099, and 1.00 ± 0.00), and bronchus (1.00 ± 0.00, 1.00 ± 0.00, and 1.00 ± 0.00). Latency was 0.23 ± 0.06 seconds, and 4D model reconstruction was completed in 8.47 seconds.

**Conclusions:**

Pulmo-SR integrates artificial intelligence, finite element method, and 3D modeling to provide a 4D deformable reconstruction of patient anatomy, offering realistic simulations for complex lung resections. Clinical validation demonstrated high accuracy, precision, and sensitivity, indicating the potential as a valuable tool in preoperative and intraoperative workflows for anatomical lung resections.

## Graphical Abstract

**Figure:**
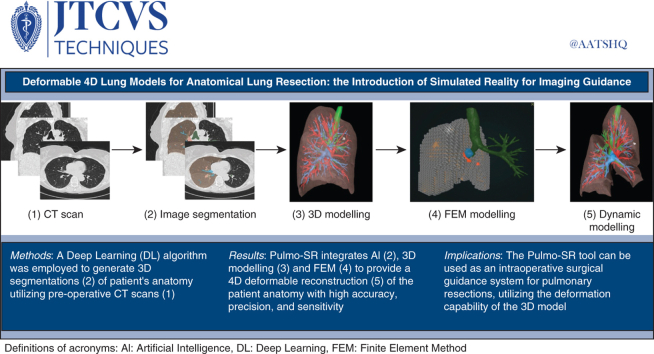

**Figure:**
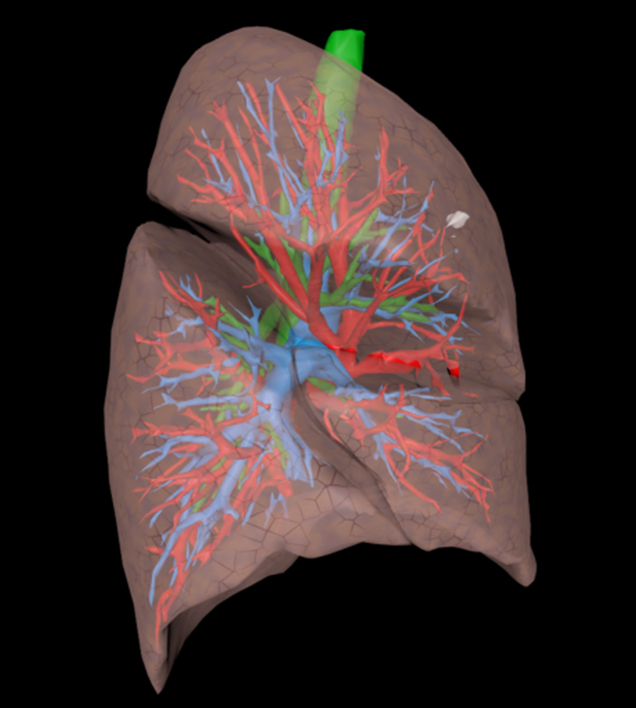
3D deformable model for pre- and intraoperative surgical guidance.

**Central Message**

Pulmo-SR uses artificial intelligence, finite element modeling, and 3- and 4-dimensional simulation to support lung surgery with anatomy visualization for planning and guidance.

**Perspective**

Pulmo-SR combines artificial intelligence, finite element modeling, and 4-dimensional simulation to potentially enhance pulmonary resections through real-time, high-precision anatomical modeling. Although not yet compared with standard imaging in outcome studies, it may improve surgical planning and intraoperative orientation by providing more individualized anatomical insight.

In the planning phase of anatomical lung resections, such as lobectomy or segmentectomy, 3-dimensional (3D) visualization has become increasingly important and is recommended by the European Society of Thoracic Surgeons expert consensus document.^1^ For patients with anatomical variations, identifying the pulmonary vessels and airways supplying the lobe or segment of interest can be challenging using conventional 2-dimensional computed tomography (CT) scans.^2^ Therefore, using patient-specific 3D models can be beneficial to prevent the surgical ligation of incorrect vessels. This approach can also lead to shorter operative times, fewer instances of bleeding, lower rates of postoperative complications, and reduced durations of postoperative drainage.^1^^,^^3^, ^4^, ^5^

Medical image segmentation, such as CT scan segmentation, is a technique used to obtain 3D reconstructions of patient anatomy.^6^ It involves partitioning a medical image into distinct regions or segments corresponding to different anatomical structures, tissues, or regions of interest.^7^ For lung surgery planning, image segmentation is typically performed using preoperatively acquired CT scans. Visualizing these 3D segmentations during the preoperative planning process helps improve the understanding of patient-specific anatomy, which can lead to better preparation and decision making regarding the extent of resection.^2^^,^^8^^,^^9^

Because manual segmentation of CT scans is time-consuming and can take several hours, artificial intelligence (AI) can help reduce the manual workload associated with this process.^10^ Several studies have applied AI to automatically segment thoracic structures such as pulmonary vessels, airways, and lung lobes.^11^, ^12^, ^13^ These automatically generated 3D segmentations can then be visualized using open-source or commercially available software. However, these 3D models are static, based on preoperative CT scans, and do not accurately reflect the patient's intraoperative anatomy, where the lungs are desufflated and continuously exposed to manual deformations by the surgeon. As a result, these models are less suitable for intraoperative guidance or surgical navigation during a procedure.

In a previously published study, we demonstrated a computational proof-of-concept dynamic lung model that enabled digital lung manipulation and deformations of the 3D models.^14^ However, this approach had several drawbacks, such as the inability to adjust mechanical tissue properties, complex and time-consuming model creation involving many manual steps, and slow simulation speeds with low frame rates that influenced the smoothness and responsiveness of motion.

In the current approach, we aim to overcome these challenges by leveraging technologies such as deep learning-based image segmentation and finite element method (FEM) physical modeling. The goal of this study is to develop Pulmo-Simulated Reality (Pulmo-SR), a tool that rapidly generates patient-specific, manually deformable lung models with minimal manual effort. This tool aims to provide ease of use for intraoperative guidance and seamless integration into the clinical workflow. In this article, we present the technical development path and evaluate both the technical performance and clinical applicability and accuracy.

## Materials and Methods

### Patient Population

In this study, CT scans (maximum slice thickness of 1 mm) and intraoperative surgical video recordings of 30 consecutive patients who underwent an anatomical lung resection (eg, lung lobectomy or segmentectomy) at the Erasmus MC (EMC), Rotterdam, the Netherlands, between January 2023 and March 2024 were used after providing written informed consent for the publication of study data, approved by the Institutional Medical Ethical Committee (MEC-2023-008 [date of approval: February 26, 2023]/MEC 2023-0397 [date of approval: August 31, 2023]). All data were anonymized and handled according to the EMC privacy guidelines.

### 3D Visualization and Dynamic Modeling

**Figure 1:**
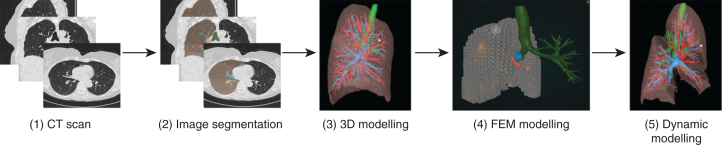
Overview of the workflow. The deep learning algorithm is used on the preoperative computed tomography scan (1) to create an automatic segmentation (2) of the pulmonary artery (*blue*), vein (*red*), airways (*green*), and lung lobes (*pink*). Utilizing 3-dimensional (3D) visualization software, a 3D model can be created (3). By dividing the 3D model into smaller tetrahedra using the finite element method (4), a dynamic deformable simulated reality 4-dimensional model can be created (5).

Preoperative CT scans were used to generate 3D segmentations of the pulmonary artery, vein, airways, and lung lobes using a deep learning algorithm, called nnUNet, an open-source framework for biomedical image segmentation.^15^ The tumor was manually delineated (labeled) by a trained expert (Q.M.) in agreement with the thoracic surgeon performing the surgery. Segmentations were saved as Neuroimaging Informatics Technology Initiative files for medical imaging and object files, a standard 3D geometry file format used to store information about the shape and structure of 3D modeling and visualization. These segmentations were visualized using 3D software (Surgical Reality). Figure 1 illustrates the workflow.

Dynamic models were created by 1 software engineer (T.K.) using the FEM,^16^ which discretizes the system into smaller elements (eg, cubes or tetrahedra). These elements together represent a FEM mesh. This allows for complex calculations and simulates physical interactions between adjacent elements. The global system behavior is modeled by aggregating results from all individual elements, with each lung lobe represented by an FEM mesh. Physical interactions between adjacent elements were modeled using constitutive material laws, with mechanical properties (eg, elasticity and density) assigned according to the type of tissue represented. These properties were tissue-specific, with distinct parameter sets for arteries, veins, bronchi, and lung parenchyma, based on values reported in the literature.^17^, ^18^, ^19^, ^20^, ^21^, ^22^

The simulation's realism was influenced by the number of elements used to subdivide the volume.^16^ More elements improved precision but increased computational load. To address this, parallelization was employed, distributing calculations across multiple processors. A balance between realism and real-time responsiveness depends on the number of FEM elements. Deformations of 3D segmented structures, like lung lobes and arteries, were mapped to FEM elements. Material parameters were adjusted for each tissue to simulate accurate deformations.^23^

To ensure real-time performance, the simulation needed minimal delay, providing instantaneous feedback to the surgeon without perceptible lag. The real-time requirement was quantified by recording the frames per second (FPS) of the simulation. The target was 90 FPS, with 60 FPS considered sufficient for similar applications.^24^ FPS was calculated by analyzing the time between frames. Deformation input, model changes, and the time taken for the system to stabilize were also recorded.

**Figure 2:**
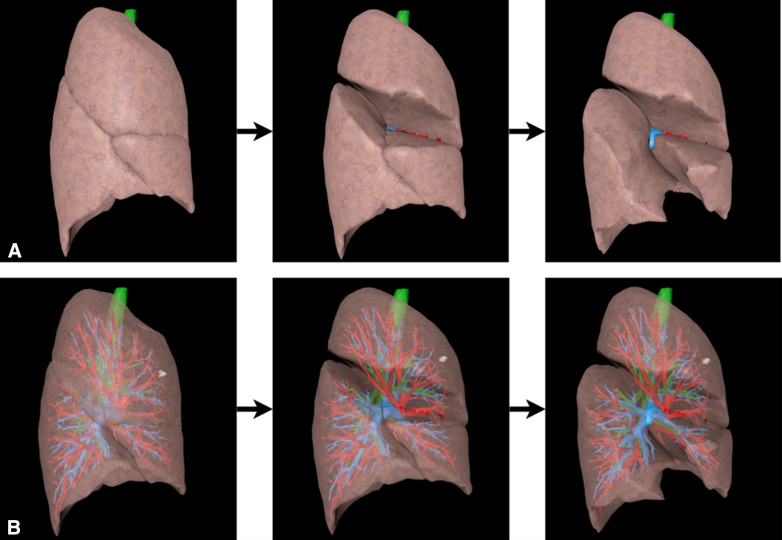
Example of the deformation applied to the deformable model (fissure view) based on the full 3-dimensional model with lung lobes (row A) and with a 50% transparency of the lung lobes (row B).

To assess the 3D models' usability, response time for five default deformations (1 per lobe) was measured. These deformations, designed to simulate lobar fissure separation, were consistently applied to each lobe, with proportional scaling based on lobe size. An example is shown in Figure 2. The simulation ran on a system with an Intel 12th Gen i7-12700H processor (Intel Corporation), 16 GB RAM, and an NVIDIA GeForce RTX 3060 GPU (NVIDIA Corporation).

### Technical Setup

**Figure 3:**
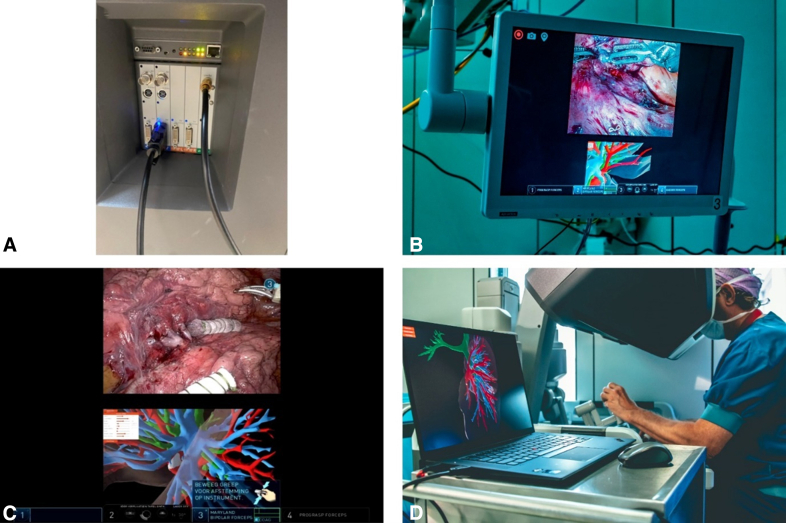
Intraoperative setup of the dynamic 3-dimensional (3D) model with the wireless mouse to manually deform the 3D models according to the intraoperative situation. A, Connection of the laptop with the DaVinci robotic console (Intuitive Surgical) using a video graphics array-to-high-definition multimedia interface cable. B, Intraoperative screen view after activating the TilePro∗. C, DaVinci console view after activating the TilePro. D, Intraoperative setup of the dynamic 3D model with the wireless mouse to manually deform the 3D model according to the intraoperative situation. ∗TilePro is a multi-input video display feature available in the DaVinci console.

The dynamic model was used intraoperatively by connecting a Lenovo ThinkPad X1 Extreme Gen 5 laptop to the DaVinci robot (Intuitive Surgical) console via a digital visual interface to high-definition multimedia interface cable. The TilePro function on the DaVinci console allowed the surgeon to view the Pulmo-SR 4D (deformable in time) models on the laptop below the intraoperative view (Figure 3). A wireless mouse enabled the surgeon to manually manipulate and deform the 4-dimensional (4D) model to match the intraoperative situation (Figure 3). The surgeon validated the model's orientation during the procedure. Intraoperative use of the 4D model is shown in Video 1.

### Clinical Evaluation

A preliminary clinical evaluation was performed by 2 surgeons from the EMC (A.M. and S.S.) to assess the relevance and usability of the 4D dynamic models. Intraoperative recordings were used to compare the automatically generated 3D model with the actual anatomy by manually adjusting the 4D model to the intraoperative situation. Accuracy (Equation 1) was calculated by comparing the 3D model to the intraoperative view of the pulmonary anatomy (artery, vein, and bronchus). Accuracy measures how often the model's predictions are correct overall.

(1)

(1) $$Accuracy=\frac{TP+TN}{TP+TN+FP+FN}$$

where true positive (TP) is the number of structures correctly identified by the AI 3D model as artery/vein/bronchus, false positive (FP) is the number of structures incorrectly identified by the AI 3D model as artery/vein/bronchus, and false negative (FN) is the number of structures that were missed by the AI 3D model as artery/vein/bronchus. True negative (TN) was described as the number of structures (artery/vein/bronchus) not identified by the AI 3D model and not visible intraoperatively. This number will always be 0 and can be neglected. Besides accuracy, also the precision (Equation 2) and sensitivity (Equation 3) were calculated for all 3 structures (artery/vein/bronchus) individually. Precision measures the proportion of true positives among all positive predictions, and sensitivity measures the proportion of actual positives identified.

(2) $$Precision=\frac{TP}{TP+FP}$$

(3) $$Sensitivity=\frac{TP}{TP+FN}$$

In most cases, the analysis was limited to the anatomical structures, arterial, venous, and bronchial, within the operative lobe or segment involved in the procedure. However, in some instances, adjacent anatomical structures from neighboring segments or lobes were also visible due to surgical exposure or retraction. Because the goal of this study was to clinically evaluate the accuracy of the 3D models, these structures were also included. Although it is true that not all distal segmental vessels are clearly visualized intraoperatively, the key segmental branches relevant for ligation and dissection were identifiable in the cases. A result of 1 indicates a 100% correct segmentation.

## Results

### Patient Characteristics

The characteristics of the 30 patients are summarized in Table 1. Most patients were women (67% [n = 20]), with a median age of 67 years (interquartile range, 59-71 years). Nineteen patients (63.3%) underwent lobectomy, and 11 (36.7%) underwent segmentectomy. Tumors were distributed across all 5 lung lobes: right upper lobe: n = 7 (23.3%), right middle lobe: n = 1 (3.3%), right lower lobe: n = 11 (36.7%), left upper lobe: n = 6 (20%), and left lower lobe: n = 5 (16.7%).

**Table 1:** Characteristics of the included patients (N = 30)

Characteristic	Result
Age (y)	67 (59-71)
Sex	
Female	20 (66.7)
Male	10 (33.3)
Histology	
Benign lesion	3 (10)
Carcinoid	3 (10)
Metastasis	2 (6.7)
Squamous cell carcinoma	5 (16.7)
Leiomyosarcoma	1 (3.3)
Adenocarcinoma	16 (53.3)
Tumor location	
RUL	7 (23.3)
RML	1 (3.3)
RLL	11 (36.7)
LUL	6 (20)
LLL	5 (16.7)
Surgery type	
Segmentectomy	11 (36.7)
Lobectomy	19 (63.3)

Values are presented as median (interquartile range) or n (%). *RUL*, Right upper lobe; *RML*, right middle lobe; *RLL*, right lower lobe; *LUL*, left upper lobe; *LLL*, left lower lobe.

### 3D Visualization and 4D Dynamic Modeling

**Figure 4:**
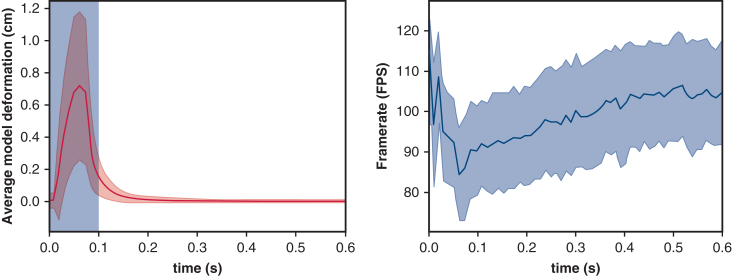
*Left*, The average deformation of the 3-dimensional model over time. The *blue* region indicates the timeframe where the input deformation is applied. The *red line* is the average deformation of all points on the 4-dimensional model and the *red* region shows the standard deviation of the displacement. *Right*, The average framerate in frames per second for all measurements shown over time. The *blue line* shows the average framerate with the standard deviation of the measurements shown with the blue region.

**Figure E1:**
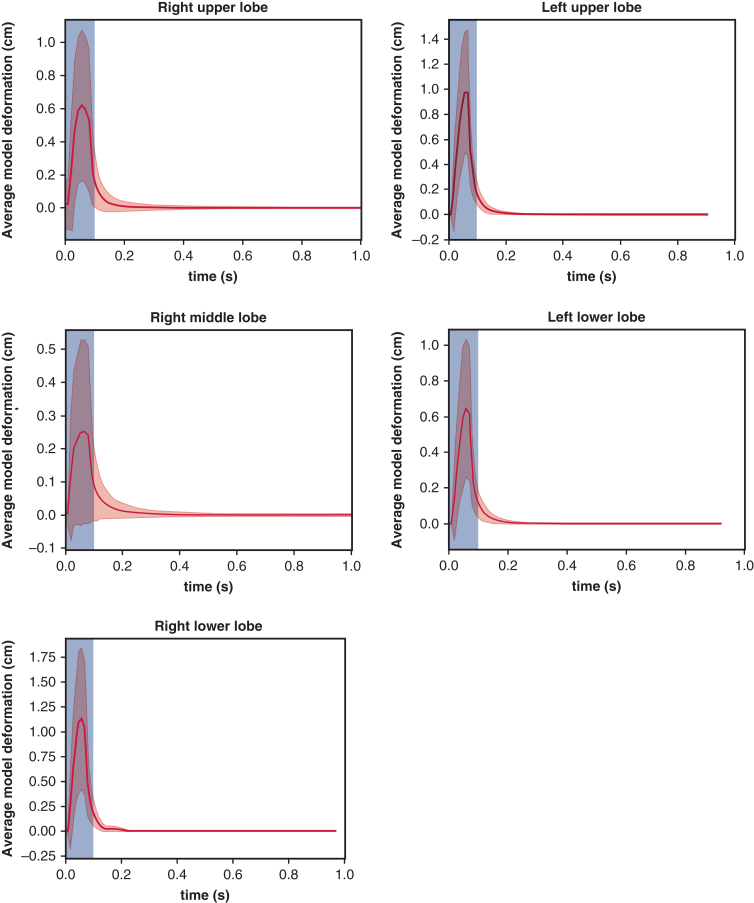
The deformation of the 3-dimensional model over time for each lobe. The *blue* region indicates the timeframe where the input deformation is applied. The *red line* is the average deformation of all points on the 4-dimensional model and the *red* region shows the standard deviation of the displacement.

**Figure E2:**
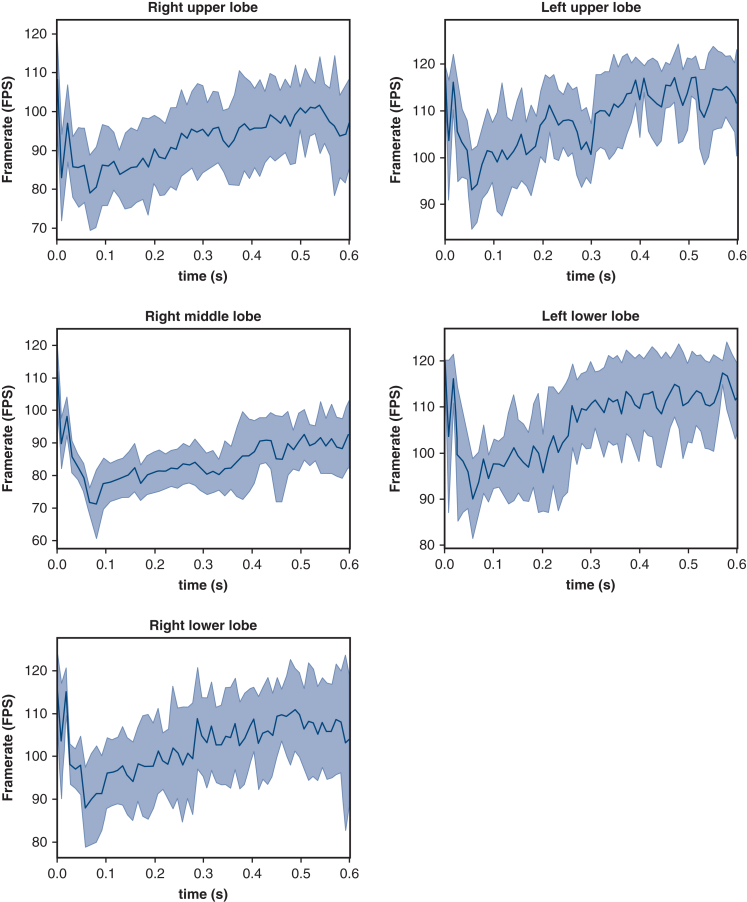
The framerate in frames per second for all measurements shown over time for each lung lobe. The *blue line* shows the average framerate with the SD of the measurements shown with the *blue* region.

The measurements per lobe are shown in Figures E1 and E2, and the average measurements are presented in Figure 4. The average number of tetrahedra in all lung models represented by a FEM mesh was 4096. Tetrahedra are 3D geometric elements composed of 4 triangular faces, commonly used in computational modeling and finite element analysis. Loading the segmentations, creating the 3D models, and setting up the 3D simulation took an average of 8.47 seconds for these 8 cases on the specified hardware configuration.

Figure 4 also presents the average model displacement over time. The deformation applied to each lobe always took 0.1 seconds. The time region of the deformation is indicated by the blue area in the figure. The average model displacement approached 0 shortly after the deformation was complete, indicating that the 4D model converged to a stable state on average 0.23 ± 0.06 seconds after the deformation stopped.

The average FPS over time is shown in Figure 4 as well. The average framerate did not drop below 85 FPS during the simulation, with occasional standard deviation spikes not falling below 70 FPS. When the average displacement and error rates were near 0, the average framerate remained steadily above 90 FPS.

### Clinical Evaluation

**Figure 5:**
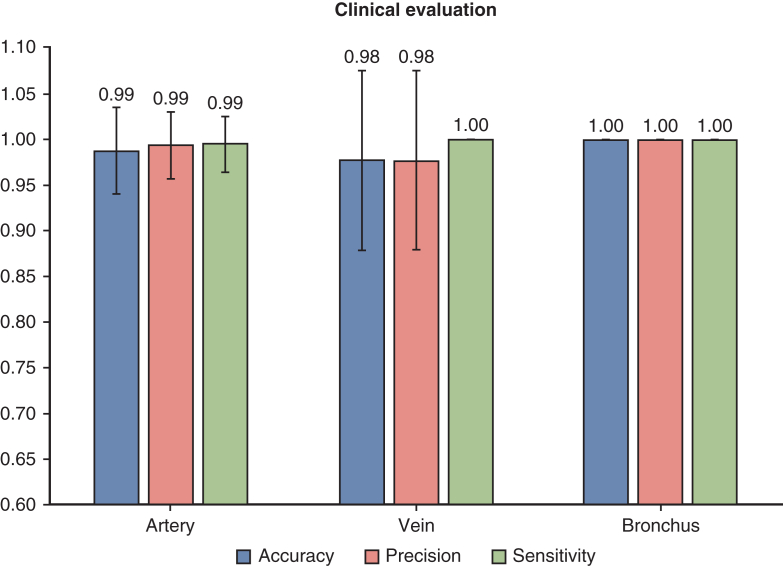
Clinical evaluation of the accuracy, precision, and sensitivity with SD for the artery, vein, and bronchus. Value represents the average scores.

The accuracy, precision, and sensitivity scores for the artery, vein, and bronchus in 30 cases were calculated using Equations ((1), (2), (3)). Figure 5 illustrates the boxplot of clinical validation scores. The mean scores for accuracy, precision, and sensitivity for the different structures were, respectively: 0.987 ± 0.047, 0.993 ± 0.037, and 0.994 ± 0.031; vein: 0.976 ± 0.099, 0.976 ± 0.099, and 1.00 ± 0.00; and bronchus: 1.00 ± 0.00, 1.00 ± 0.00, and 1.00 ± 0.00.

An overview of the total number of arterial, venous, and bronchial branches visible intraoperatively and predicted by the AI algorithm for each case is presented in Table 2. According to Table 2, 1 case (case 15) showed an incorrect (false positive) artery segmentation, 1 case (case 23) showed a missing (false negative) artery, and 2 cases (cases 11 and 18) showed incorrect (false positive) vein segmentations. All bronchus segmentations were correct based on intraoperative recordings.

**Table 2:** Overview of the clinical evaluation

Case	Artery video	Artery AI	Vein video	Vein AI	Bronchus video	Bronchus AI
1	4	4	3	3	1	1
2	4	4	3	3	x	x
3	2	2	4	4	3	3
4	2	2	3	3	1	1
5	3	3	4	4	x	x
6	5	5	1	1	2	2
7	4	4	2	2	2	2
8	4	4	5	5	1	1
9	6	6	3	3	1	1
10	4	4	3	3	3	3
11	x	x	**1**	**2**	x	x
12	6	6	3	3	1	1
13	1	1	4	4	2	2
14	3	3	3	3	3	3
15	**4**	**5**	4	4	3	3
16	3	3	3	3	1	1
17	4	4	2	2	1	1
18	4	4	**6**	**7**	1	1
19	3	3	2	2	2	2
20	5	5	2	2	1	1
21	3	3	3	3	2	2
22	5	5	3	3	2	2
23	**6**	**5**	3	3	1	1
24	2	2	x	x	2	2
25	3	3	x	x	2	2
26	3	3	2	2	1	1
27	5	5	x	x	3	3
28	3	3	4	4	2	2
29	4	4	3	3	3	3
30	3	3	3	3	3	3

For every case, the artery, vein, and bronchus intraoperative was compared with the AI output. The numbers indicate the number of structures (vessels or airways) visible in the video or visible in the 3-dimensional model created by the AI. Numbers in boldface type indicate a deviating value. *AI*, Artificial intelligence; *x*, missing value.

## Discussion

3D visualization technology is emerging as a tool to support surgeons during complex procedures.^25^ Translating 2-dimensional CT images to 3D intraoperative anatomy can be challenging.^2^ To address this, we introduced a technique for both preoperative planning and intraoperative imaging assistance during lung resections, such as lobectomies and segmentectomies. This provides surgeons with an interactive tool that aids in identifying critical anatomical structures, such as pulmonary vessels and airways.

Although many studies evaluate AI algorithms using metrics like the Dice score, we focused on the clinical benefits and accuracy of 3D models for surgical use.^26^, ^27^, ^28^ By employing a deformable 4D model, we simulated realistic surgical scenarios, making the models effective for real-time surgical assistance.

Through accuracy, precision, and sensitivity analyses, combined with intraoperative recordings, we demonstrated the clinical relevance of these 3D models. However, not all structures were visible during surgery. For example, in segmentectomy cases, the ligated vein and parenchymal borders were not visualized (Table 2). Additionally, very small vessels, absent from the AI model's training database, were missing. In 1 case, the AI identified an extra vessel, although the origin differed from the actual anatomy. The model mistakenly showed 2 origins, whereas the intraoperative findings showed a single origin with a bifurcation further distally.

Because AI algorithms are unlikely to achieve perfect segmentation accuracy, it is essential to evaluate how inaccuracies may influence surgical decision making and patient outcomes.^29^ In our study, the model demonstrated a low but non-0 rate of vascular misclassification. Although these did not lead to intraoperative complications, their presence underscores the importance of defining what level of accuracy is clinically acceptable. This includes weighing the frequency of errors against their potential to cause harm. Simulation tools should be used to support, rather than replace, surgical expertise, with final intraoperative decisions grounded in anatomical confirmation. Future studies should systematically assess the consequences of missing or misclassified structures, particularly in larger, multicenter settings involving multiple surgeons and patients, to establish safety thresholds and validation standards necessary for clinical implementation. A limitation of this study is the manual deformation of the 3D model. To implement this deformable model in the daily workflow, automatic deformation based on real-time intraoperative conditions is needed. We are developing an AI algorithm to recognize lung lobe orientations during surgery,^30^^,^^31^ which, when integrated into the Pulmo-SR platform, could allow automatic model adjustments in real time, enhancing surgical guidance. AI could also serve as an augmented reality tool, overlaying the deformable 3D model onto the surgeon's view, improving navigation during procedures.^32^^,^^33^

The 3D models are based on preoperative CT scans of insufflated lungs, which do not accurately reflect the desufflated state during surgery.^34^ Simulating desufflation is challenging due to the complex mechanical properties of tissues like lung parenchyma, vessels, and bronchi. Previous work by Villard and colleagues^35^ modeled lung behavior using FEM, focusing on elasticity, stiffness, and pressure distribution to simulate lung deformation. Future advancements should integrate desufflation simulation to enhance the realism of these 3D models. Although we acknowledge that critical vascular events are uncommon, their potential influence is substantial. Pulmo-SR is not primarily intended to prevent rare complications, but rather to improve workflow by increasing confidence in segmental anatomy, supporting margin assessment, and reducing intraoperative uncertainty. These factors, in combination, may contribute to more efficient and consistent surgical performance over time. As outlined in the European Society of Thoracic Surgeons guidelines, determination of arterial segmental borders is preferred. On this platform, the segmental borders align with these recommendations and may help improve clinical outcomes by providing greater certainty in defining them. However, these clinical assessments still require further clinical validation.

In this study, important variables such as body mass index, operative time, complications, and patient outcomes were not analyzed because the platform was not used to guide surgical strategies. Our focus was limited to clinical assessment and the development of a dynamic deformable model, which may serve as a tool for surgical guidance. Future research that incorporates these clinical parameters could provide a more complete picture of the added value of this simulation platform. Because this technology enables the creation of an accurate representation of the intraoperative anatomy, it could represent an important step toward image-guided surgery. These dynamic models were already implemented in the first proof-of-concept augmented reality lobectomy, where they helped reduce uncertainties during complex procedures. Such applications could further expand the role of augmented reality in surgical decision making. A sufficiently advanced augmented reality system could also facilitate more lung-sparing procedures, such as subsegmentectomies, while reducing the learning curve for surgeons. Because the model accounts for anatomical deformation, it provides a more accurate representation of intraoperative conditions. This, in turn, can accelerate the understanding of patient-specific anatomy and support faster surgical training.

Many publications on image segmentation use the Dice score to evaluate the accuracy of AI models in generating 3D segmentations from new CT scans. The Dice score quantifies the overlap between predicted and ground truth segmentations, ranging from 0 (no overlap) to 1 (perfect overlap). However, combining this technical metric with clinical validation allows for a more comprehensive assessment of the usability of 3D segmentations and deformable models in clinical settings.^36^ Although technical evaluation metrics, such as the Dice score that our model achieved (mean Dice between 0.91 and 0.92), may sometimes be lower, the clinical usability of the 3D reconstruction can still be high.^37^ However, achieving this Dice score requires using a maximum CT scan slice thickness of 1 mm. In our clinical validation, we achieved an overall accuracy, precision, and sensitivity of 0.99, 0.99, and 0.98, respectively, suggesting that the Dice score alone may not fully capture the clinical usefulness of these 3D models.

An important point to note is the integration of the Pulmo-SR models into the TilePro of the robotic console. This might suggest that the platform is only applicable for robotic-assisted thoracoscopic surgery procedures. However, the software can also be used on a laptop for video-assisted thoracoscopic surgery procedures by placing the laptop next to the operating room screen to create a side-by-side visualization. This makes the software platform equally useful for video-assisted thoracoscopic surgery procedures.

However, this study was limited to only 30 cases, making it primarily a pilot study. Thus, no definitive conclusions can be drawn regarding the models' accuracy and reliability at this stage. Although initial results are promising, further clinical validation with a larger, more heterogeneous dataset, including more surgeons, centers, and patients, is necessary to assess the general clinical usability of the Pulmo-SR models.

Although the anatomical relationships between pulmonary arteries, veins, and bronchi remain constant, the potential advantage of a deformable model lies in simulating the way these structures appear during surgical manipulation and intraoperative displacement. Prior studies, such as clinical validation of 3D dynamic models,^37^ have shown that automated vessel segmentation can provide a strong foundation for 3D planning in lung segmentectomy. Building on this, our study explored whether adding deformability can improve the surgeon's ability to anticipate technical challenges, such as vessel exposure or bronchial displacement, that are not readily appreciated by static reconstruction alone. The deformable models were developed with the long-term goal of enabling augmented reality superimposition during thoracic surgery, as proposed by Sadeghi and colleagues.^32^ Each component of the development process was scientifically evaluated and tested before clinical implementation to ensure reliability and accuracy. The technical tests described in this article were therefore conducted to further refine and enhance the realism of the dynamic model. In addition, clinical usability was assessed through a pilot evaluation in the clinical setting, in line with recent advances in automated 3D planning and vessel segmentation.^37^ Furthermore, ongoing development aims to incorporate lung collapse, providing a more representative configuration of the lung relative to the pathological specimen. Such functionality may allow more accurate predictions of resection margins and spatial relationships during segmentectomy. Although this work represents an early step toward clinical validation, the findings suggest that interactive and dynamic models may complement existing 3D planning tools by offering a more intuitive bridge between preoperative imaging and intraoperative anatomy.

The simulation's framerate and deformation measurements show that the model converges to a stable state within 0.23 seconds, indicating a real-time response to the surgeon's input. Overall, the simulation maintains a framerate above the required 60 FPS, with convergence occurring in 0.23 seconds. The important to note that several factors influence simulation performance. The number of elements in the FEM mesh is critical; increasing the number can reduce performance, while decreasing it may improve performance but compromise accuracy. For consistency, the number of elements was kept constant across both performance and clinical measurements. Hardware configuration is also a key factor. The laptop used, valued at approximately €2500, influences performance, and variations in hardware (higher or lower) will correspondingly influence the simulation results.

## Conclusions

In this study, we have combined AI, the FEM, and 3D visualization to develop a new interactive tool, Pulmo-SR, which can be used as an intraoperative surgical guidance system for pulmonary resections, utilizing the deformation capability of the 3D models. The clinical validation shows high accuracy, precision, and sensitivity scores. This may aid in pre- and perioperative anatomical assessment and potentially support decision making during complex lung resection, although further validation is needed.

## Conflict of Interest Statement

Mr Mank is a part-time employee at Surgical Reality. Mr Kieft is a full-time employee at Surgical Reality. Dr Maat is a clinical advisor and shareholder in Surgical Reality. Dr Sadeghi is cofounder and CMO of Surgical Reality. All other authors reported no conflicts of interest.

The *Journal* policy requires editors and reviewers to disclose conflicts of interest and to decline handling or reviewing manuscripts for which they may have a conflict of interest. The editors and reviewers of this article have no conflicts of interest.

## Supplementary Data

Video 1 An example of the use of the Pulmo-SR 4D model. Video available at: https://www.jtcvs.org/article/S2666-2507(25)00482-1/fulltext.

## References

[1] Brunelli A., Decaluwe H., Gonzalez M. (2023). European society of thoracic surgeons expert consensus recommendations on technical standards of segmentectomy for primary lung cancer. Eur J Cardiothorac Surg.

[2] Sadeghi A.H., Maat A.P.W.M., Taverne Y.J.H.J. (2021). Virtual reality and artificial intelligence for 3-dimensional planning of lung segmentectomies. J Thorac Cardiovasc Surg Tech.

[3] Ji Y., Zhang T., Yang L. (2021). The effectiveness of three-dimensional reconstruction in the localization of multiple nodules in lung specimens: a prospective cohort study. Transl Lung Cancer Res.

[4] Saji H., Inoue T., Kato Y. (2013). Virtual segmentectomy based on high-quality three-dimensional lung modelling from computed tomography images. Interact Cardiovasc Thorac Surg.

[5] Kato H., Oizumi H., Suzuki J., Hamada A., Watarai H., Sadahiro M. (2017). Thoracoscopic anatomical lung segmentectomy using 3D computed tomography simulation without tumour markings for non-palpable and non-visualized small lung nodules. Interact Cardiovasc Thorac Surg.

[6] Pham D.L., Xu C., Prince J.L. (2000). Current methods in medical image segmentation. Annu Rev Biomed Eng.

[7] Suetens P., Bellon E., Vandermeulen D. (1993). Image segmentation: methods and applications in diagnostic radiology and nuclear medicine. Eur J Radiol.

[8] Bakhuis W., Sadeghi A.H., Moes I. (2023). Essential surgical plan modifications after virtual reality planning in 50 consecutive segmentectomies. Ann Thorac Surg.

[9] Sadeghi A.H., Mathari S.E., Abjigitova D. (2022). Current and future applications of virtual, augmented, and mixed reality in cardiothoracic surgery. Ann Thorac Surg.

[10] Gleeson F., Revel M.P., Biederer J. (2023). Implementation of artificial intelligence in thoracic imaging-a what, how, and why guide from the European Society of Thoracic Imaging (ESTI). Eur Radiol.

[11] Nam J.G., Witanto J.N., Park S.J., Yoo S.J., Goo J.M., Yoon S.H. (2021). Automatic pulmonary vessel segmentation on noncontrast chest CT: deep learning algorithm developed using spatiotemporally matched virtual noncontrast images and low-keV contrast-enhanced vessel maps. Eur Radiol.

[12] Nadeem S.A., Hoffman E.A., Sieren J.C. (2021). A CT-based automated algorithm for airway segmentation using freeze-and-grow propagation and deep learning. IEEE Trans Med Imaging.

[13] Park J., Yun J., Kim N. (2020). Fully automated lung lobe segmentation in volumetric chest CT with 3D U-Net: validation with intra- and extra-datasets. J Digit Imaging.

[14] Bakhuis W., Max S.A., Nader M. (2023). Video-assisted thoracic surgery S7 segmentectomy: use of virtual reality surgical planning and simulated reality intraoperative modelling. Multimed Man Cardiothorac Surg.

[15] Isensee F., Jaeger P.F., Kohl S.A.A., Petersen J., Maier-Hein K.H. (2021). nnU-Net: a self-configuring method for deep learning-based biomedical image segmentation. Nat Methods.

[16] Bathe K. (2006).

[17] Bernal M., Nenadic I., Urban M.W., Greenleaf J.F. (2011). Material property estimation for tubes and arteries using ultrasound radiation force and analysis of propagating modes. J Acoust Soc Am.

[18] Wang J.Y., Mesquida P., Pallai P., Corrigan C.J., Lee T.H. (November 2011). Dynamic properties of human bronchial airway tissues. http://arxiv.org/abs/1111.5645.

[19] Kwon S., Yang W., Moon D., Kim K.S. (2020). Comparison of cancer cell elasticity by cell type. J Cancer.

[20] Skacel P., Bursa J. (2022). Poisson’s ratio and compressibility of arterial wall—improved experimental data reject auxetic behaviour. J Mech Behav Biomed Mater.

[21] Al-Mayah A., Moseley J., Velec M., Brock K.K. (2009). Sliding characteristic and material compressibility of human lung: parametric study and verification. Med Phys.

[22] Butler J.P., Nakamura M., Sasaki H., Sasaki T., Takishima T. (1986). Poisson’s ratio of lung parenchyma and parenchymal interaction with bronchi. Jpn J Physiol.

[23] Sicard D., Haak A.J., Choi K.M., Craig A.R., Fredenburgh L.E., Tschumperlin D.J. (2018). Aging and anatomical variations in lung tissue stiffness. Am J Physiol Lung Cel Mol Physiol.

[24] Va H., Choi M.H., Hong M. (2021). Real-time cloth simulation using compute shader in Unity3D for AR/VR contents. Appl Sci.

[25] Zhang X., Yang D., Li L. (2024). Application of three-dimensional technology in video-assisted thoracoscopic surgery sublobectomy. Front Oncol.

[26] Tan W., Zhou L., Li X., Yang X., Chen Y., Yang J. (2021). Automated vessel segmentation in lung CT and CTA images via deep neural networks. J Xray Sci Technol.

[27] Zhang H., Zhang M., Gu Y., Yang G.Z. (2023). Deep anatomy learning for lung airway and artery-vein modeling with contrast-enhanced CT synthesis. Int J Comput Assist Radiol Surg.

[28] Zulfiqar M., Stanuch M., Wodzinski M., Skalski A. (2023). DRU-Net: pulmonary artery segmentation via dense residual U-network with hybrid loss function. Sensors (Basel).

[29] Kenig N., Monton Echeverria J., Muntaner Vives A. (2024). Artificial intelligence in surgery: a systematic review of use and validation. J Clin Med.

[30] He K., Zhang X., Ren S., Sun J. Deep residual learning for image recognition. https://www.cv-foundation.org/openaccess/content_cvpr_2016/papers/He_Deep_Residual_Learning_CVPR_2016_paper.pdf.

[31] Czempiel T., Paschali M., Keicher M. (2020). Medical Image Computing and Computer Assisted Intervention – MICCAI 2020.

[32] Sadeghi A.H., Mank Q., Tuzcu A.S. (2024). Artificial intelligence-assisted augmented reality robotic lung surgery: navigating the future of thoracic surgery. J Thorac Cardiovasc Surg Tech.

[33] Doornbos M.J., Peek J.J., Maat A.P.W.M. (2024). Augmented reality implementation in minimally invasive surgery for future application in pulmonary surgery: a systematic review. Surg Innov.

[34] Dawda S., Camara M., Pratt P., Vale J., Darzi A., Mayer E. (2019). Patient-specific simulation of pneumoperitoneum for laparoscopic surgical planning. J Med Syst.

[35] Villard P.F., Beuve M., Shariat B., Baudet V., Jaillet F. (2005). Simulation of lung behaviour with finite elements: influence of bio-mechanical parameters. Proceedings of the Third International Conference on Medical Information Visualisation—BioMedical Visualisation. London, United Kingdom.

[36] Li X., Zhang S., Luo X. (2023). Accuracy and efficiency of an artificial intelligence-based pulmonary broncho-vascular three-dimensional reconstruction system supporting thoracic surgery. EBioMedicine.

[37] Mank Q.J., Thabit A., Maat A.P.W.M. (2025). Artificial intelligence-based pulmonary vessel segmentation: an opportunity for automated three-dimensional planning of lung segmentectomy. Interdisc Cardiovasc Thorac Surg.

