# Supplementary figures and images for: Deformable lung models for anatomical lung resections: The introduction of simulated reality for imaging guidance
Source: JTCVS Tech. 2025 Nov 10;35:102147. doi: 10.1016/j.xjtc.2025.10.022 (PMC12881800; doi:10.1016/j.xjtc.2025.10.022)

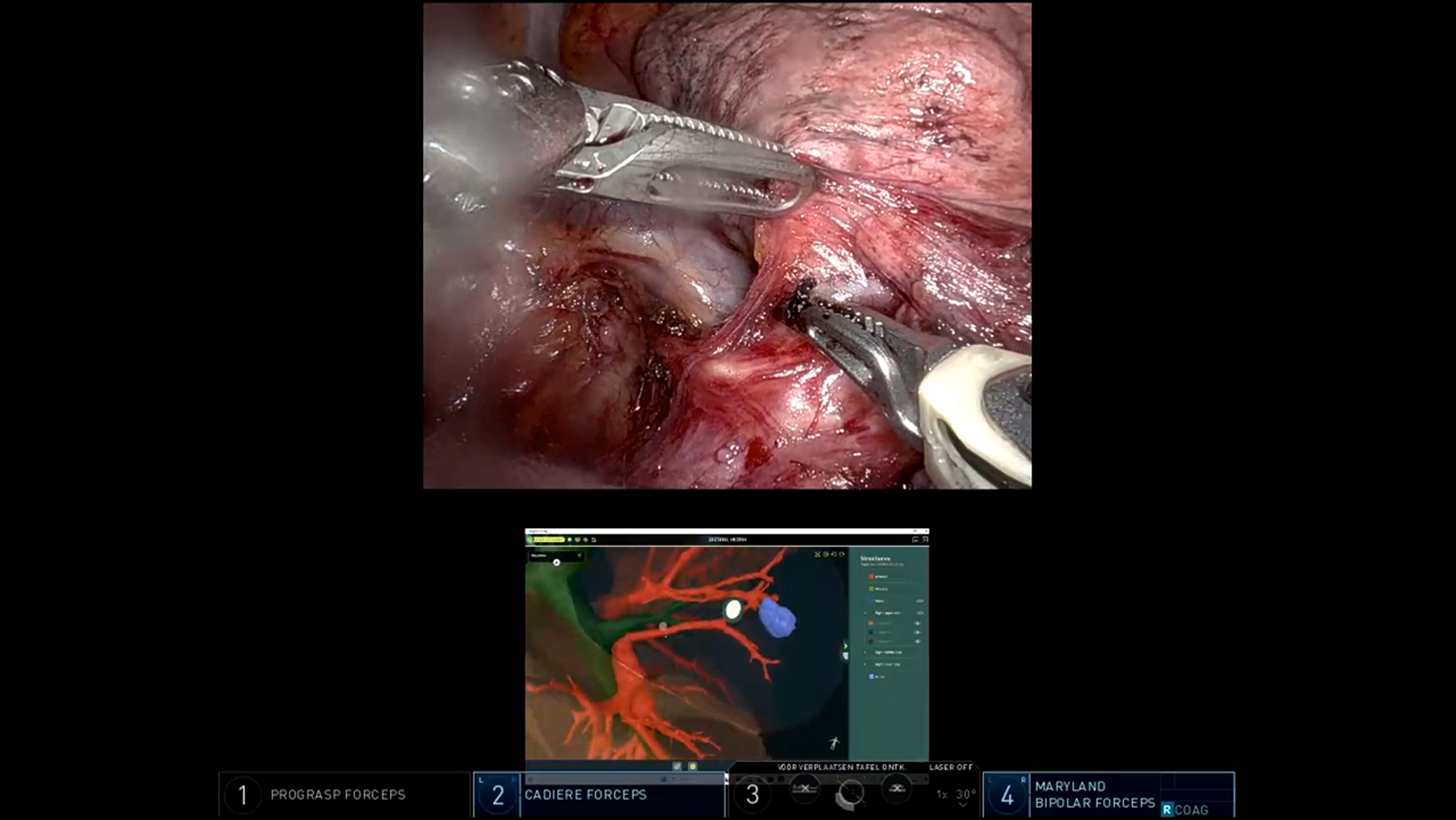

Supplement: Video 1 — An example of the use of the Pulmo-SR 4D model. Video available at: https://www.jtcvs.org/article/S2666-2507(25)00482-1/fulltext. [file fx2.jpg]
